# Application of Multiprocessing Technology of Motion Video Image Based on Sensor Technology in Track and Field Sports

**DOI:** 10.1155/2022/4430742

**Published:** 2022-02-11

**Authors:** Shaofeng Xu, Junmeng Chen

**Affiliations:** Sangmyung University, Seoul 03016, Republic of Korea

## Abstract

To improve the accuracy of track and field sports feature recognition, this paper combines sensor technology to improve the motion video image multiprocessing technology and gives the basic principles of image registration. Moreover, this paper chooses a model based on projection transformation. When using a high-speed linear CCD, only the image information on the finish line is collected. Unlike the previous high-speed area CCD cameras that can capture runway information, linear CCDs are used to collect only the image information on the finish line, and the data is collected and processed through sensor technology. The research shows that the application effect of the motion video image multiprocessing technology based on sensor technology in track and field sports proposed in this paper has good practical effects.

## 1. Introduction

Images are objective and true image data obtained from the real world through various observation and measurement systems and various observation and measurement methods. It can be directly received by the human eye and generates a signal in the human brain to make the human visually perceive the actual information. After scientific research, it has been confirmed that about 80% of the information obtained by humans comes from the human visual system, which means that about three-quarters of the information obtained by people is in the form of images [1]. This is the most basic qualitative for the word image. Images can convey a lot of information and are not susceptible to interference from other factors. In addition, it can be expressed in the most true, direct, and objective manner, and the received information can be processed twice at the fastest speed through the widely used multimedia information technology nowadays. On this basis, we discover the relevant information about the athletes we need in training, so as to ensure that coaches and the researchers responsible for monitoring and investigating the athletes' training situation can obtain the most direct, intuitive, and highly referential training information of athletes and can also use this as a basis to guide athletes on how to train in the follow-up process [2]. Therefore, the related technology of image processing has been welcomed and favored by coaches and athletes for a long time. This method can play a very huge role in the process of researching sports [3].

Video images are obtained by people using various tools to observe the objective world in different forms and methods. It can directly or indirectly act on the human eyes and produce visual perception entities, that is, static pictures or dynamics observed by the human eyes. Video. Scientific research and statistics show that about 75% of the information that humans obtain from the outside world comes from the visual system, which is obtained from images. After the two-dimensional or three-dimensional analysis of the sports technology video image, the image or data information of the athlete's technical performance is obtained, and then, the technical training level is evaluated through scientific analysis and evaluation. All of this is based on the video image. Image processing technology can most intuitively monitor and evaluate athletes' technical movements, and it can also stimulate athletes' interest in learning and improving sports technology the most. At the same time, the sports technology video is carried out under the condition of no interference to the athletes. It directly obtains the real technical data of the athletes at the competition and training site, which can best meet the requirements of sports training and the real situation of the competition. Therefore, video image processing technology is one of the most effective scientific research methods and has a wide range of application prospects.

This article combines sensor technology to improve the multiprocessing technology of sports video images, researches the process of track, and field sports and builds an intelligent track and field analysis system, which provides a reference for the subsequent improvement of the effect of track and field sports.

## 2. Related Work

The development of modern multimedia technology has enabled computers to intensively manage and process the media that disseminate information, such as text, graphics, sound, and images, so that modern educational technology with computers as the core has become a key experimental field for school teaching reform in the new era. Physical education is an important part of school education and school physical education. Teaching methods and methods should also be continuously enriched and developed, and its reform is imperative [4]. The characteristic of multimedia teaching is that it is suitable for expressing visualized or simple logical thinking teaching content based on concrete images. Using multimedia computers to integrate text, graphics, images, and videos can present more information to students in a short period of time, which cannot be accomplished by traditional teaching methods in the same time period [5]. There are many media in physical education. The use of media is sufficient, complete, diverse, and efficient, which is conducive to improving the quality of teaching and is conducive to the learning and mastery of sports movements [6].

Multimedia technology is the result of the comprehensive development of related technologies, such as microelectronics, computer technology, and communication technology. Therefore, it is a highly comprehensive high-tech. With the development and improvement of multimedia technology itself, its concept is also constantly developing, the meaning it covers is also constantly extending, and its related fields are constantly expanding [7].

After the 1990s, with the development of computer technology, the continuous upgrading of hardware and software, the continuous improvement of multimedia technology, the continuous improvement of the support capacity of the PC system platform, the continuous improvement of compression and decompression technology and coding technology, and the development of network technology, it solves the storage, transmission, and performance of multimedia information [8]. The multimedia system has the ability to comprehensively process sound, graphics, and text, especially the problem of processing full-motion video information and interactive video applications. Video images are obtained by people using various tools to observe the objective world in different forms and methods. It can directly or indirectly act on the human eye and produce visual perception entities, that is, static pictures or dynamics observed by human eyes. The video [9]. After the two-dimensional or three-dimensional analysis of the sports technology video image, the image or data information of the athlete's technical performance is obtained, and then, the technical training level is evaluated through scientific analysis and evaluation, all of which are based on the video image [10]. From the perspective of the development of video image acquisition and processing methods, it has experienced from early film photography to infrared spot and AV signal video recording and then digital DV signals that are widely used at present. From the perspective of the development of image acquisition speed, it has experienced low-speed video and normal-speed video to high-speed video; from analog signal to digital signal, from low-resolution video to the development of high-precision images, from two-dimensional measurement analysis to three-dimensional measurement analysis; in the display after the picture is collected, from black and white images to color images; in terms of technical feedback, through field video collection, laboratory video collection, analysis, and post-exercise Feedback, up to the current on-site video collection, on-site video technology analysis and processing, on-site rapid feedback, feedback time is greatly shortened [11]; in the way of video feedback, from single person single video technical data feedback, to multi-person and multi-video comparison Feedback, video overlay feedback, etc.; in terms of obtaining motion parameters, it has also experienced the development from complex to simple, from long-term feedback to rapid feedback on the sports scene, from complex technical processing to simple technical rapid processing. All of this is closely connected with the rapid development of modern science and technology, especially computer technology [12]. Scientific research and statistics show that about 75% of the information that humans obtain from the outside world comes from the visual system, which is also obtained from images [13]. After the two-dimensional or three-dimensional analysis of the sports technology video image, the image or data information of the athlete's technical performance is obtained, and then, the technical training level is evaluated through scientific analysis and evaluation, all of which are based on the video image [14]. Image processing technology can most intuitively monitor and evaluate athletes' technical movements, and it can also stimulate athletes' interest in learning and improving sports technology the most. At the same time, the sports technology video is carried out under the condition of no interference to the athletes. It directly obtains the real technical data of the athletes at the competition and training site, which can best meet the requirements of sports training and the real situation of the competition. Therefore, video image processing technology is one of the most effective scientific research methods and has a wide range of application prospects [15].

## 3. Multiprocessing Technology of Track and Field Video Image

The geometric transformation model of track and field sports images uses the matrix form in the mathematical model to express the change relationship between two track and field sports images, such as translation, rotation, and zoom transformation. In fact, the mathematical model is used to express the geometric transformation model of the track and field sports image, which is convenient for the simulation and simulation of the algorithm. Before the two track and field sports images are fused, the corresponding coordinate points of the same objects in the two track and field sports images need to be found. When shooting, the two track and field sports images to be registered may be affected by the experimental environment. There are some changes. Different mathematical models should be selected for different situations to describe the geometric transformations of the track and field sports images. Some common transformations are translation, rotation, affine, projection and so on. Among them, the first two are simple transformations, which are uniformly divided into rigid body transformations.

The rigid body transformation can only represent the translation and rotation transformation between the coordinates of two track and field sports images. Formula (1) represents the mathematical expression of rigid body transformation [16].

(1) $$\begin{array}{c} \left(\begin{array}{c} x^{\prime }\\ y^{\prime }\\ 1 \end{array}\right)=\left(\begin{array}{ccc} \cos\theta & -\sin\theta & m_{2}\\ \sin\theta & \cos\theta & m_{5}\\ 0 & 0 & 1 \end{array}\right)\left(\begin{array}{c} x\\ y\\ 1 \end{array}\right). \end{array}$$

Rigid body transformation is the simplest transformation among the three transformations, and there are only three degrees of freedom. *m*_2_, *m*_5_ represents the horizontal offset and vertical offset between two track and field sports images. 0 represents the rotation angle between two athletic images, (*x*′, *y*′) is the coordinate after transformation, and (*x*, *y*) is the coordinate of the original image.

Affine transformation can not only describe the translation and rotation transformations between the pixels of two track and field sports images but also describe their scaling transformations. Formula (2) expresses the mathematical expression of affine transformation [17].

(2) $$\begin{array}{c} \left(\begin{array}{c} x^{\prime }\\ y^{\prime }\\ 1 \end{array}\right)=\left(\begin{array}{ccc} m_{0} & m_{1} & m_{2}\\ m_{3} & m_{4} & m_{5}\\ 0 & 0 & 1 \end{array}\right)\left(\begin{array}{c} x\\ y\\ 1 \end{array}\right). \end{array}$$

It is not difficult to see that (*x*′, *y*′) are the transformed coordinates, and (*x*, *y*) are the coordinates in the original image. There are six in the transformation matrix. *m*_0_, *m*_1_, *m*_3_, and *m*_4_ represent one of the two track and field sports images. Rotation and zoom between the two, *m*_2_ and *m*_5_, represent the amount of translation. It can be known from the number of unknowns that at least three pairs of corresponding feature points are needed to obtain the transformation matrix.

**Figure 1:**
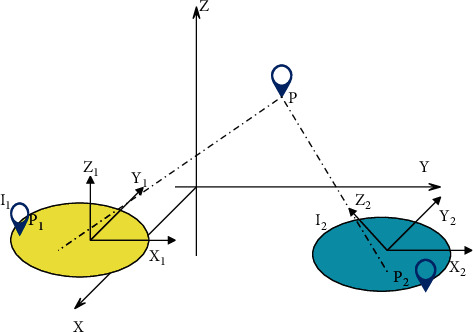
Coordinates mapped during actual shooting.

The projection transformation is closer to actual life. The shapes of the two track and field sports images before and after the transformation are basically unchanged, but the original parallel line segments no longer remain parallel. After repeated experimental verification, this paper found that the effect of projection transformation is significantly better than that of other transformations. Therefore, this paper adopts the projection transformation, and there are eight degrees of freedom in the projection transformation. According to the values of these eight degrees of freedom, people can accurately know what transformations have occurred in the two track and field sports images. The models and principles of these three transformations have been introduced previously. Next, this paper combines the projection transformation selected in this paper and the coordinates mapped in real shooting to do a detailed introduction.  Figure 1 shows the coordinates mapped during the actual shooting.

As shown in Figure 1, where *P* (*X*, *Y*, *Z*) is any point in the natural scene, *C*_1_ and *C*_2_ are two different imaging sensors with translation and rotation transformations. *P*_1_, *P*_2_ is the imaging coordinates of the unified scenic spot *Р* taken by the two different imaging sensors, respectively, marked as *x*_1_=(*X*_1_, *Y*_1_, *k*_1_)^*T*^, *x*_2_=(*X*_2_, *Y*_2_, *k*_2_)^*T*^, so formula (3) can be derived [18].

(3) $$\begin{array}{c} x_{1}=V_{1}R_{1}T_{1}P,\\ x_{2}=V_{2}R_{2}T_{2}P. \end{array}$$

Among them, *T*_*i*_, *V*_*i*_, *R*_*i*_(*i*=1,2), respectively, represent the translation, scaling, and rotation matrix of the imaging coordinate system of the acquisition device relative to the world coordinate system. Formula (4) shows the relationship between *x*_1_ and *x*_2_:

(4) $$\begin{array}{c} x_{2}=V_{2}R_{2}T_{2}T_{1}{}^{-1}R_{1}{}^{-1}V_{1}{}^{-1}x_{1}. \end{array}$$

*M*=*V*_2_*R*_2_*T*_2_*T*_1_^−1^*R*_1_^−1^*V*_1_^−1^, and there is *x*_2_=*Mx*_1_, where *M* is a 3 × 3 matrix, which is generally written as follows:

(5) $$\begin{array}{c} x_{2}=\left(\begin{array}{c} X_{2}\\ Y_{2}\\ k_{1} \end{array}\right)=\left(\begin{array}{ccc} m_{0} & m_{1} & m_{2}\\ m_{3} & m_{4} & m_{5}\\ m_{6} & m_{7} & 1 \end{array}\right)\left(\begin{array}{c} X\\ Y\\ k \end{array}\right), \end{array}$$

(6) $$\begin{array}{c} X_{2}=\frac{m_{0}X_{1}+m_{1}Y_{1}+m_{2}}{m_{6}X_{1}+m_{7}Y_{1}+1}, \end{array}$$

(7) $$\begin{array}{c} Y_{2}=\frac{m_{3}X_{1}+m_{4}Y_{1}+m_{5}}{m_{6}X_{1}+m_{7}Y_{1}+1}. \end{array}$$

The previously mentioned formula shows that if the coordinates of a point in the actual scene captured and imaged by two different sensors are *x*_1_ and *x*_2_, a 3 × 3 transformation matrix can be used to express the transformation relationship between two track and field sports images. According to the transformation matrix, people can calculate the specific values of translation, rotation, and scaling between two track and field sports images. The projection transformation model has 8 degrees of freedom mi (*i* = 0, 1, 2,…, 7), where *k* is the scaling parameter. The perspective transformation is that the original parallel line segments no longer remain parallel, but the original straight-line segments are still straightline segments.

The detection of feature points is to detect the feature points of the differential Gaussian pyramid of track and field sports images of different scales. The so-called feature points are points that do not change under the changes of translation, rotation, and scaling, that is, the scale invariance factor. In fact, the difference pyramid is used to refine the track and field motion image and then detect the feature points. The differential Gaussian pyramid of track and field sports images is defined as [19]

(8) $$\begin{array}{c} D\left(x,y,k\sigma \right)=\left(G\left(x,y,k\sigma \right)-G\left(x,y,\sigma \right)\right)⊗I\left(x,y\right),\\ G\left(x,y,k\sigma \right)=\frac{1}{2\pi \sigma^{2}}e^{\left(-\left(x^{2}+y^{2}\right)/2\sigma^{2}\right)}. \end{array}$$

Among them, *D*(*x*, *y*, *kσ*) represents the differential Gaussian pyramid of the track and field sports image with a scale of 0 under the coefficient *k*, *D*(*x*, *y*, *σ*) represents the Gaussian pyramid of the scale of *G*(*x*, *y*, *kσ*), 1(*x*, *y*) represents the original track and field sports image, and ⊗ represents the convolution between them. *σ* is the scale factor, *G*(*x*, *y*, *kσ*) represents the Gaussian function of scale, and (*x*, *y*) is the coordinate of the point on the track and field sports image. In order to find the characteristic points on the difference pyramid, take any point on the track and field sports image of the difference pyramid as the center point in the 3 × 3 window, and then take the 3 × 3 window of the upper and lower layers of the difference pyramid corresponding to this layer. Whether the value of the center point is greater than the value of 26 points in its neighboring or corresponding points in the upper and lower windows is compared. If it is, then, the point is regarded as the maximum point, that is, the feature point of interest; otherwise, it is not, and the feature point detected on the track and field sports image is obtained.

The matching method of sift in Lowe is mainly based on Euclidean distance. However, the threshold value selected each time is different, and the specific threshold value is determined mainly based on repeated experiments. The matching of two descriptors *D*1 and *D*2 is when the distance between only two descriptors is *T* times (threshold) not greater than the distance between *D*1 and other descriptors, it is considered as a corresponding matching point as follows:

(9) $$\begin{array}{c} d\left(D1,D2\right)\times 1.5<d\left(D1,\text{others}\right). \end{array}$$

The Chinese meaning of surf is fast and robust. After the sift algorithm was researched, later generations found that although the sift algorithm is very stable, it runs very slowly. The Sift algorithm uses a differential Gaussian pyramid, while the surf algorithm uses an approximation of the determinant of the Hessian matrix. Formula (10) represents a Hessian matrix with point *x* at scale *σ*.

(10) $$\begin{array}{c} H\left(x,\sigma \right)=\left[\begin{array}{cc} L_{xx}\left(x,\sigma \right) & L_{xy}\left(x,\sigma \right)\\ L_{xy}\left(x,\sigma \right) & L_{yy}\left(x,\sigma \right) \end{array}\right]. \end{array}$$

Among them, *L*_*xx*_(*x*, *σ*) represents the convolution of the function value of the second-order Gaussian differential ∂^2^/∂*x*^2^*g*(*σ*) with the track and field moving image I at point *x*. *L*_*xy*_(*x*, *σ*) represents the convolution of the function value of the second-order Gaussian differential ∂^2^/∂*x*  ∂*yg*(*σ*) with the track and field moving image *I* at point *x*. *L*_*yy*_(*x*, *σ*) represents the convolution of the function value of the Gaussian second-order differential ∂^2^/∂*y*^2^*g*(*σ*) with the track and field moving image *I* at the point *x*.

**Figure 2:**
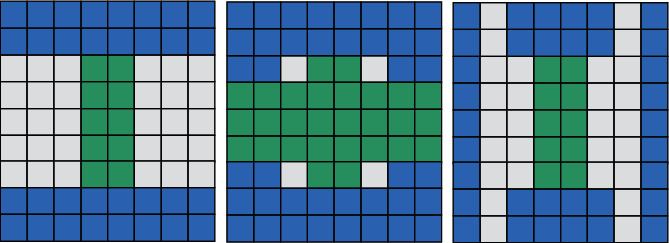
Box filter.

People can use the corresponding template to convert the result of the convolution into the form of a box filter, as shown in Figure 2:

If a Gaussian second-order differential function with a standard deviation of 1.2 is used for filtering, a 9 × 9 template is selected to convolve and detect track and field moving images. Formula (12) represents the simplified formula for the determinant value of the Hessian matrix:

(11) $$\begin{array}{c} \text{Det}\left(H_{\text{approx}}\right)=D_{xx}D_{yy}-{\left(0.9D_{xy}\right)}^{2}. \end{array}$$

**Figure 3:**
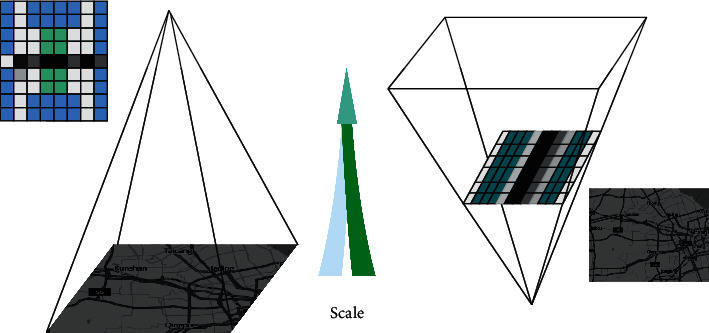
Sift pyramid and surf pyramid.

From Figure 3, we can see the difference between the pyramid constructed with the sift algorithm and the pyramid constructed with the surf algorithm [20]. That is, when the sift algorithm is used to construct the pyramid, the size of the track and field sports image is continuously changed after sampling, but the size of the template is fixed. However, when the surf algorithm is used to construct the pyramid, the size of the track and field motion image is unchanged, but the size of the filter is constantly changing, thus constructing an integral function on the original track and field motion image and speeding up the running speed of the surf algorithm.

**Figure 4:**
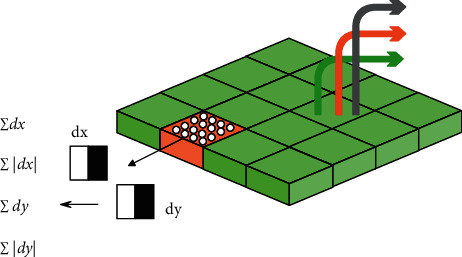
Schematic diagram of Harr wavelet characteristics.

In the surf algorithm, the horizontal and vertical eigenvalues of the Harr wavelet in the neighborhood of the feature point are counted. The range of the neighborhood is to rotate at a certain angular interval in a sector with an arc center angle of 60 degrees in the circle of. Then, the sum of the eigenvalues of the Harr wavelet in the sector are, respectively, counted, and the main direction of the characteristic point is determined according to the largest statistical value. Then, according to the obtained main direction, draw a square that is consistent with the direction near the feature point. The square is divided into 4 × 4 small areas, a total of 16 areas, and the eigenvalues of the Harr wavelet of 25 pixels in each small area are calculated. The characteristic value includes the cumulative value of the horizontal direction, the cumulative value of the vertical direction, the cumulative value of the absolute value of the horizontal characteristic value, and the cumulative value of the absolute value of the vertical characteristic value. The schematic of the process is shown in Figure 4:

In this way, the cumulative value of four directions in 16 regions is formed, a total of 64-dimensional vectors, and the sift algorithm has a total of 128-dimensional vectors, so the amount of calculation is reduced by half, which greatly accelerates the matching speed of the algorithm. For the fusion of two cameras that use different fields of view (FOV) and resolution, the first thing that needs to be realized is the transformation relationship between all the track and field sports images involved. Therefore, it is necessary to spatially remap each track and field sports image to a common coordinate. This can be achieved by a simple registration method. The geometric registration formulates several key assumptions. First, the two cameras are assumed to be concentrated at the same point. Therefore, the center of each track and field sports image should match spatially before any registration. Second, it assumes that when the two cameras are synchronized, the frames that produce the same scene are related to time. Finally, it is assumed that the slight difference in the actual camera position between the scenes viewed by the two cameras at a certain distance is negligible [21].

**Figure 5:**
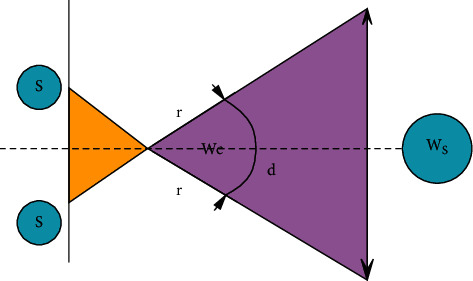
Registration specifications.

The final hypothesis can be obtained from Figure 5. Two planes appear in this picture, the length of Wc represents the sensor plane and the length of Ws represents the scene plane. The distance S is related to the offset of the two cameras installed. The scene at the sensor level is quantized into *N* discrete pixels, and the width of each pixel is described as follows:

(12) $$\begin{array}{c} \text{Pixel Widt}=\frac{W_{c}}{N}. \end{array}$$

Therefore, the plane of the scene is also quantized into *N*, and the following describes the discrete part of the pixel width:

(13) $$\begin{array}{c} \text{Scene Widt}=\frac{W_{s}}{N}=2d\;\tan\left(\gamma \right). \end{array}$$

In order for a significant difference in the two frames of the two offset cameras to appear, there must be at least 1 SceneWidth. Therefore, as long as the value of S is less than one pixel shift, there is no difference in the distance between the two cameras in the final track and field sports image.

(14) $$\begin{array}{c} S<\frac{d\;\tan\left(\gamma \right)}{N}. \end{array}$$

The distance *d* is solved by the rearrangement (15) algorithm, and the result is

(15) $$\begin{array}{c} d>S\times \frac{N}{\tan\left(\gamma \right)}. \end{array}$$

Arbitrarily, if *S* is equivalent to a value close to 10 cm, that is, the camera is installed at a distance of 10 cm, 240 pixels are used, and the field of view is less than 90 degrees, and the minimum distance *d* can be found. The result of this calculation shows that as long as the two cameras are concentrated to a distance greater than 2.40 meters, no offset can be seen in the two track and field sports images.

**Figure 6:**
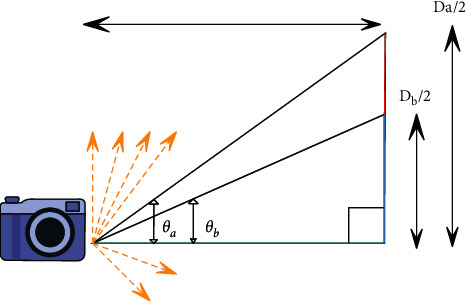
Geometric registration.

In all other track and field sports images, the track and field sports images called “source” are sampled to achieve the same resolution as the benchmark track and field sports images. The first step in this process involves taking a useful part of the base geometry, as shown in Figure 6. Angle 0 is equivalent to half of a specific angle of view. The relationship between the angle and the length of this side is expressed as follows [22]:

(16) $$\begin{array}{c} \tan\left(\theta \right)=\frac{D}{2D_{t}}. \end{array}$$

Since the *D*_*t*_ of the two cameras is the same, this value can be normalized to get the relative length of the track and field sports image, and the result can be expressed by formula (17). Since this formula uses the relative height and width of each frame, each frame can be found:

(17) $$\begin{array}{c} D=2\;\tan\left(\theta \right). \end{array}$$

These distances are used to remove any areas that will not appear in the two frames. In order to achieve this, the relative distance of each track and field sports image must be compared. No matter which frame contains a larger distance, it must be cropped to a frame with a smaller size. Since the resolution of each camera may change, this difference is converted into pixels. This value indicates how many rows or columns must be removed, which is expressed by the following:

(18) $$\begin{array}{c} \text{Num Croppe Npixels}\left[1-\frac{D_{\text{Min}}}{D_{\text{Max}}}\right]. \end{array}$$

## 4. Application of Multiprocessing Technology of Motion Video Image Based on Sensor Technology in Track and Field Sports

**Figure 7:**
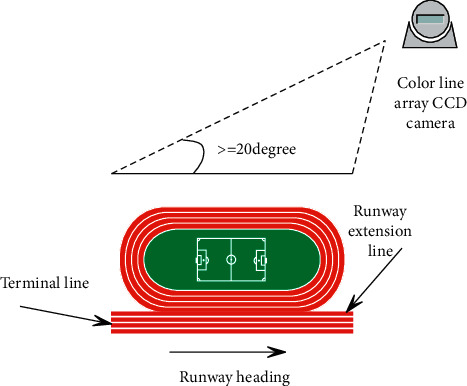
Schematic diagram of camera placement and runway identification.

This research uses high-speed linear CCD to collect only the image information on the finish line. Unlike the previous high-speed area CCD camera that can capture runway information, the linear CCD only collects the image information on the finish line. Next, we need to solve the runway problem. If the track cannot be resolved, the matching of the athlete's performance will be affected to a certain extent. In order to solve this problem, the finish line can be drawn as a white line, and the runway line can be extended to the finish line with a black line, so that the CCD acquisition system can collect the black line information of the runway. Since there is runway information on each frame of image, in this way, the line of the runway appears on the image composed of the linear CCD images of each frame, and the rest of the runways are white, which is easy to distinguish. The schematic diagram of camera placement and runway identification is shown in Figure 7.

**Figure 8:**
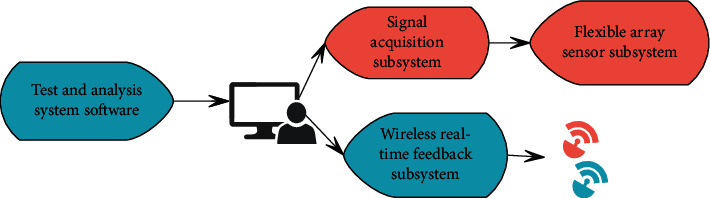
Block diagram of system modules.

This system is composed of upper plastic layer, sensor unit, row lead, column lead, lower plastic layer, sensor acquisition node, field bus unit, digital track module, field bus, computer, and wireless headset. It is characterized by the structure of a flexible array sensor based on a screen printing process, and a plurality of sensor units are placed between the upper plastic layer and the lower plastic layer for protection. The sensor unit is connected by row leads and column leads to form a sensor acquisition node. Moreover, multiple sensor collection nodes are connected with the lower plastic layer and the upper plastic layer, and the field bus unit to form a large-area flexible digital track module as a training platform, which is fixed on the ground. In the large-area flexible digital racetrack module, the row leads of each row of sensor units and the column leads of each sensor unit are connected to the field bus unit. Furthermore, it is connected to the computer through the field bus to complete the transfer of information collection to the computer, and the wireless headset receives the feedback information from the computer through the wireless communication network. The block diagram of the system modules is shown in Figure 8.

**Figure 9:**
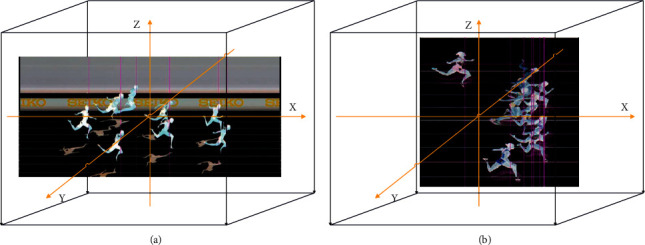
Case images of multiprocessing technology for track and field sports videos. (a) Case image 1. (b) Case image 2.

According to the system proposed in this paper, a case image of the multiprocessing technology of track and field sports video is obtained, as shown in Figure 9.

**Figure 10:**
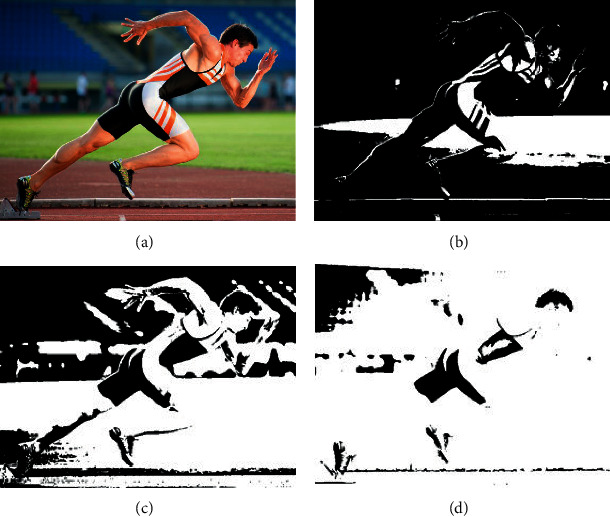
The processing effect of the motion video image multiprocessing technology. (a) Original image. (b) Grayscale processing. (c) Noise removal. (d) Feature recognition.

Through the multiprocessing technology of the track and field sports video for image processing, the image processing effect is shown in Figure 10.

Based on this analysis, the application effect of this system is evaluated, and the results shown in Table 1 are obtained.

From the previously mentioned research, we can see that the multiprocessing technology of motion video image based on sensor technology proposed in this paper has good practical effects in track and field sports.

## 5. Conclusion

With the continuous improvement of sports technology and computer performance, the intensity of track and field events is also increasing. During the track and field competition, the accuracy of the athlete's starting movement can enable the athlete to obtain the best speed in the shortest time, which directly affects the competition performance, so it has become the focus of research in the field of sports teaching. In the actual training process, due to the differences in the physical conditions and specific physical abilities of different athletes, some athletes have more wrong starting movements, and their ability to master the correct starting movements is poor. In this state, how to effectively conduct in-depth observation and judgment of athletes' starting movements and use certain effective means to prevent and correct them has become the main problem that needs to be solved urgently in this field, which has attracted the attention of many experts and scholars in this field. Therefore, many good research results have been obtained. This article combines sensor technology to improve the multiple processing technology of sports video images, researches the process of track and field sports, and builds an intelligent track and field analysis system, which provides a reference for the subsequent improvement of the effect of track and field sports.

## Figures and Tables

**Table 1:** Application effect of multiprocessing technology of motion video image based on sensor technology in track and field sports.

NO	Effect	NO	Effect
1	94.16	19	92.58
2	91.70	20	83.51
3	90.10	21	86.67
4	86.47	22	88.12
5	85.25	23	88.74
6	86.94	24	91.57
7	93.57	25	86.19
8	92.25	26	93.19
9	94.83	27	94.85
10	92.51	28	84.16
11	83.51	29	84.31
12	90.22	30	86.10
13	88.03	31	86.67
14	83.64	32	83.94
15	83.63	33	86.66
16	94.98	34	84.46
17	94.08	35	91.56
18	90.37	36	85.77

## Data Availability

The labeled dataset used to support the findings of this study are available from the corresponding author upon request.
